# The Role of Plant Progesterone in Regulating Growth, Development, and Biotic/Abiotic Stress Responses

**DOI:** 10.3390/ijms231810945

**Published:** 2022-09-19

**Authors:** Hua Li, Lulu Chen, Hongyu Chen, Ruili Xue, Yuexia Wang, Jianbo Song

**Affiliations:** 1 College of Life Science, Henan Agricultural University, Zhengzhou 450002, China; 2 College of Bioscience and Bioengineering, Jiangxi Agricultural University, Nanchang 330045, China

**Keywords:** progesterone, metabolism, growth and development, biotic/abiotic stress

## Abstract

Progesterone is a steroid hormone that performs important functions in mammals. However, studies on its physiological functions in plants have gradually increased in recent years. Therefore, this review summarizes the regulatory functions of progesterone on plant growth and development, as well as its response to stress. Moreover, the plant metabolic processes of progesterone are also discussed. Overall, progesterone is ubiquitous in plants and can regulate numerous plant physiological processes at low concentrations. Since progesterone shares similar characteristics with plant hormones, it is expected to become a candidate for plant hormone. However, most of the current research on progesterone in plants is limited to the physiological level, and more molecular level research is needed to clarify progesterone signaling pathways.

## 1. Introduction

Progesterone is a steroid hormone that is synthesized in the ovaries, placenta, and adrenal glands. Although it is often considered an important female reproductive hormone, progesterone is also involved in endometrial cancer [1], breast cancer [2], central nervous system development [3], brain injury, and various neurological diseases [4,5,6]. The physiological functions and mechanisms of progesterone in mammals have therefore been studied extensively. However, adequate information regarding the physiological functioning of progesterone in plants is still lacking, which may be due to the question of whether progesterone is indeed ubiquitous in plants or not. For a long time, progesterone was thought to exist only in animals; however, the presence of progesterone was first detected in apple seeds via layer and gas-chromatography in 1968, with the progesterone levels being around 500 ng·g^−1^ [7]. Since then, researchers have gradually explored and recognized progesterone in plants. Progesterone was detected at 0.08 μg·g^−1^ in the pollen of *Pinus nigra* by radioimmunoassay (RIA) [8]. Progesterone levels were detected in dry mature wood, needles, and bark of *Pinus taeda* at 15.5, 3.85, and 1.19 μg·g^−1^, respectively [9]. Simons and Grinwich [10] found that progesterone was present in 80% of the plants they examined (128 species from over 50 families). Progesterone has also been detected in seven dicots (including *Arabidopsis*) and two monocots (including *Oryza sativa*) by Iino et al. [11]. Moreover, both studies confirmed that progesterone levels differed not only between species, but also between different tissues and organs, where the progesterone content ranged between 3–1600 ng·g^−1^. Progesterone was also subsequently found in *Digitalis purpurea*, *Nicotiana tabacum*, *Inula helenium*, and *Juglans regia*, and the progesterone concentrations in these species were comparable to previous studies [12,13]. It is therefore currently accepted that progesterone is ubiquitous in plants at low levels. Progesterone has continued to be recognized as research output has increased, which has demonstrated its important physiological functions in plants. Therefore, to better understand progesterone in plants, we summarize its metabolism and receptors, as well as its functions in regulating plant growth, development, and biotic/abiotic stress responses.

## 2. Metabolism of Progesterone in Plants

**Figure 1:**
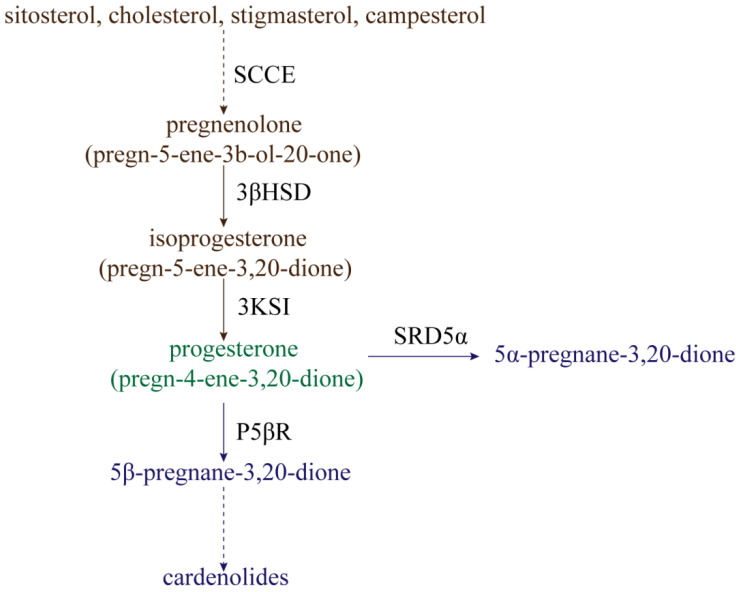
The synthesis and reduction processes of progesterone in plants. SCCE: side-chain-cleaving enzymes; 3βHSD: Δ^5^-3β-hydroxysteroid dehydrogenase; 3KSI: Δ^5^-Δ^4^-ketosteroid isomerase; P5ΒR: progesterone 5β-reductase; SRD5α: progesterone 5α-reductase.

The processes of progesterone synthesis (Figure 1) are studied by administering ^3^H or ^14^C-labeled precursors. By treating *Digitalis lanata* with ^14^C-labeled sitosterol, it was found that sitosterol could be converted into progesterone [14]. Research on leaf homogenates of *Cheiranthus cheiri* showed that progesterone can be derived from cholesterol [15]. Additionally, stigmasterol and campesterol can be catalyzed to produce progesterone by side-chain-cleaving enzymes (SCCE) at a low rate [16,17]. Therefore, sitosterol, cholesterol, stigmasterol, and campesterol are often considered to be the main precursors of progesterone synthesis in plants [18]. In mammals, side-chain-cleavage of these precursors is accomplished by SCCE (P450scc and Cyp11A1) [19,20]. However, the nature of SCCE in plants remains unclear; this is covered in detail in Lindemann’s review [21]. In addition to precursor substances, a progesterone synthesis pathway intermediate also occurs, namely, pregnenolone (pregn-5-ene-3b-ol-20-one). Pregnenolone can be converted to isoprogesterone (pregn-5-ene-3,20-dione) by Δ^5^-3β-hydroxysteroid dehydrogenase (3βHSD), whereafter isoprogesterone is isomerized to progesterone (pregn-4-ene-3,20-dione) under the action ofΔ^5^-Δ^4^-ketosteroid isomerase (3KSI) [21,22]. Unlike SCCE, 3βHSD and 3KSI activities have been confirmed in plants, with 3βHSD specifically having been explored extensively [21].

Progesterone can be reduced for use in secondary metabolism in two ways (Figure 1). One metabolic pathway is the reduction of progesterone to 5β-pregnane-3,20-dione by progesterone 5β-reductase (P5βR), whereafter 5β-pregnane-3,20-dione is further converted into cardenolides (such as digitoxigenin, digoxigenin, and gitoxigenin) under the catalyzing activities of various enzymes [23,24,25,26]. Cardenolides, without exception, possess a 5β-configuration instead of a 5α-configuration, and P5βR is therefore a key enzyme in the metabolism of progesterone to cardenolides. Klein et al. [27] identified two genes encoding progesterone 5β-reductase in *Digitalis lanata*, named *DlP5βR1* and *DlP5βR2*. After down-expression of these two genes by RNA interference (RNAi) technology, shoot cardenolide content decreased significantly. The gene encoding progesterone 5β-reductase in *Arabidopsis* was identified as *VEP1* (*At4g24220*) [28]; overexpression of the *Arabidopsis VEP1* in *Digitalis purpurea* could thus significantly increase cardenolide production. Compared with non-transgenic plants, digitoxin and digoxin content could increase up to 3.8-fold and 2.2-fold in transgenic plants cultivated in vitro, respectively [29]. Research has continually confirmed that P5βR is not only present in cardenolide-producing plants, but is also highly conserved in cardenolide-free angiosperms [30]. Therefore, P5βR might not only participate in cardenolide synthesis, but might actually have other physiological functions. For example, *P5βR2* in *Digitalis purpurea* was significantly induced by wounding, heat shock, cold shock, and salt stress [31]. Therefore, P5βR might potentially mediate abiotic stress. However, few functional studies exist regarding the P5βR abiotic stress response, and much research is still needed.

The other way in which progesterone is reduced is by conversion into 5α-pregnane-3,20-dione under the action of progesterone 5α-reductase (SRD5α) [32]. The *Arabidopsis* protein DET2 shares about 40% sequence identity with SRD5α in mammals, and has been determined to have the activity of steroid 5α-reductase. The reason for this is that, when DET2 is expressed in human embryonic kidney 293 cells, DET2 can catalyze the 5α-reduction of several steroids, including progesterone, testosterone, and androstenedione [33]. The 5α-reductase (LeDET2) identified in tomato (*Solanum lycopersicum*) has 76% homology with the DET2 protein in *Arabidopsis thaliana*. Similar to AtDET2, it is active on the substrates progesterone, testosterone, and rostenedione, but has a very low reducing activity on campestenone [34]. Two separate 5α-reductase activities were also detected in the calli and leaves of *Solanum malacoxylon*, which can perform 5α-reduction with progesterone and campestenone as substrates, but not testosterone and androstenedione [35]. DET2 might therefore catalyze different substrates in different plants, but progesterone reduction by DET2 remains ubiquitous in plants.

## 3. Research on Progesterone Receptors in Plants

Mammals have multiple progesterone receptors (PR), such as intracellular receptors, membrane-associated progesterone receptor component 1 (PGRMC1), and membrane progesterone receptors (mPR), which can mediate various physiological processes by activating downstream genes or proteins [4]. In *Arabidopsis*, a putative membrane steroid binding protein 1 (MSBP1) was identified, which can bind progesterone and other molecules (such as 5-dihydrotestosterone, 24-epi-brassinolide, and stigmasterol) with different in vitro affinities, and among them, MSBP1 has the highest affinity with progesterone [36]. Iino et al. [11] soon discovered two homologous genes of *AtMSBP1* in *Arabidopsis*, *AtMSBP2* and *AtSBP* (steroid binding protein), and cloned *OsMSBP1*, *OsMSBP2*, and *OsSBP* in rice. Furthermore, progesterone-binding membrane proteins were found to be widely distributed in plants by aligning and analyzing EST date in a variety of plants [11]. Tissue expression analysis showed that *MSBP1*, *MSBP2*, and *SBP* were expressed in various tissues [11], whereas *AtMSBP1* was weakly expressed in roots and difficult to detect in mature flowers and siliques, and that the expression level of *MSBP1* could be significantly suppressed by darkness [36].

**Figure 2:**
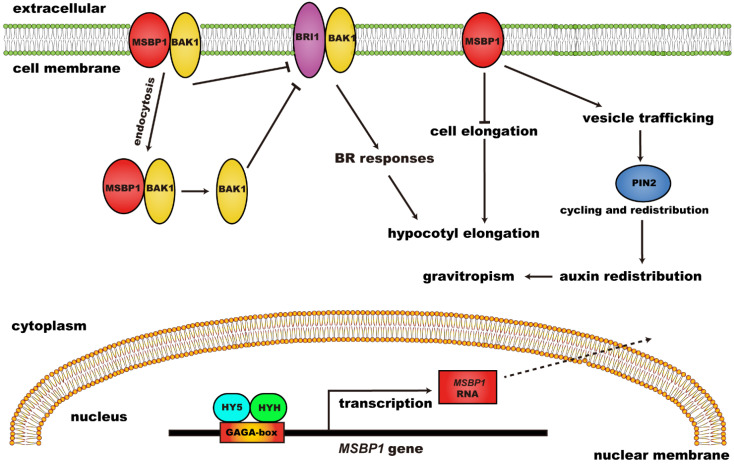
The regulatory function and mechanism of MSBP1 on hypocotyl growth and gravitropism. MSBP1: membrane steroid binding protein 1; BRI1: brassinosteroid-insensitive 1; BAK1: BRI1 associated receptor kinase 1; BR: brassinosteroid; HY5: long hypocotyl 5; HYH: HY5 homolog.

Further studies revealed that *MSBP1* can regulate hypocotyl growth and stimulate the anti-gravitropism and gravitropism of hypocotyl and roots, respectively (Figure 2). *MSBP1* overexpressing transgenic and antisense transgenic plants showed shorter and longer hypocotyl, respectively, compared to WT. The regulation of hypocotyl growth by *MSBP1* was related to its regulation of cell-elongation-related genes. *MSBP1* overexpression decreased the expression of *Exp1*, thus inhibiting cell elongation and hypocotyl growth [36]. Further studies revealed that *MSBP1* inhibits hypocotyl growth by negatively regulating brassinosteroid (BR) signaling. MSBP1 could interact with BAK1 (BRI1 associated receptor kinase 1) to promote BAK1 endocytosis, thereby reducing BR response [37]. Additionally, the upstream regulators of *MSBP1* have been found through electrophoretic mobility shift assay (EMSA) and chromatin IP (ChIP) assay. HY5 (Long Hypocotyl 5) and HYH (HY5 Homolog) can bind to the GAGA-box in the promoter to activate the transcription of *MSBP1*, thereby inhibiting the elongation of the hypocotyl [38]. Moreover, *MSBP1* was found to stimulate root gravitropism and antigravitropism of the hypocotyl by regulating auxin redistribution [39].

Progesterone also has specific binding sites in the membrane and cytoplasm of wheat (*Triticum aestivum*) cells. In non-vernalized and vernalized wheat cell membrane extracts, the specific binding of progesterone was 31.0 and 18.1 fmol/mg protein, respectively. However, the specific bindings of progesterone were 4.9 and 21.3 fmol/mg protein in the non-vernalized and vernalized wheat cell cytosolic fractions, respectively [40]. Janeczko et al. [41] confirmed that specific progesterone binding sites exist in the wheat cell membrane and cytoplasm, and the number of binding sites varies with differing drought-resistant varieties and water conditions. This suggests that steroid binding proteins are present in wheat, and that the membrane and cytoplasm content of steroid binding proteins varies significantly with vernalization and drought treatment. Unfortunately, the genes encoding for steroid binding proteins in wheat have not yet been identified.

## 4. The Regulation of Progesterone on Plant Growth and Development

**Figure 3:**
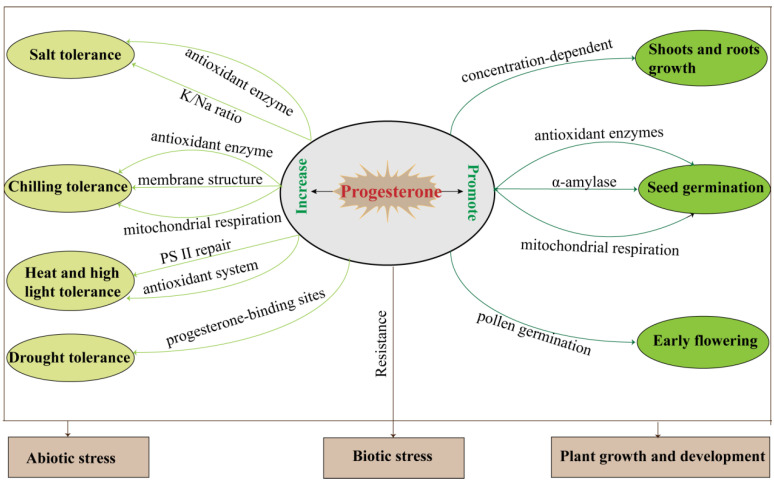
The summary of plant progesterone regulation during growth, development, and biotic/abiotic stress responses.

In addition to the above-mentioned, progesterone may be involved in hypocotyl growth and root gravitation through its receptor *MSBP1* [36,38,39], and the application of exogenous progesterone significantly affects plant shoot and root growth, seed germination, and reproductive development (Table 1, Figure 3).

### 4.1. Shoot and Root Growth

The regulation of progesterone on plant growth is concentration-dependent. For example, low progesterone concentrations (0.01–1 μM) stimulate hypocotyl elongation, while high concentrations (100 μM) inhibit hypocotyl growth in *Arabidopsis* [11]. A high progesterone concentration (0.25 μg/plant) promotes shoot growth and inhibits root growth in sunflower (*Helianthus annuus*), while a low progesterone concentration (0.1 μg/plant) induces the opposite [42]. Numerous progesterone concentrations (10^−4^–10^−15^ M) can promote chickpea (*Cicer arietinum*) shoot and root growth, with 10^−4^ M having the best effect [43]. Progesterone also affects tissue culture. Progesterone concentrations of 10^−4^ and 10^−5^ mM improve shoot formation from cotyledons explants and callus formation of sainfoin (*Onobrychis sativa*), respectively [44]. The responded embryogenic callus (REC) and regenerable callus (RE) induction from mature wheat embryos varied under different progesterone concentrations. The maximum REC rate (75%) was obtained with 10^−8^ mM progesterone treatment, but the REC rate decreased significantly under the 10^−6^ and 10^−4^ mM progesterone; 10^−6^ mM progesterone could increase RE, while RE was inhibited at the concentrations of 10^−8^ and 10^−4^ mM [45]. These results suggest that shoots and roots are generally growth-promoted at low concentrations of progesterone and growth-inhibited at high concentrations, but that optimal concentrations vary between species and at different growth stages. This feature is very similar to the regulation of plant growth by auxin and BR. Studies have confirmed that there is a certain relationship between progesterone and BR signaling (discussed in Section 7), but whether there is a link between progesterone and auxin signaling has not been explored, which is worthy of attention and discussion.

### 4.2. Seed Germination

Progesterone can significantly accelerate the rate of chickpea seed germination, which is accompanied by increased α-amylase, superoxide dismutase (SOD), and catalase (CAT) activities, but decreased seed malondialdehyde (MDA) content [46]. It was also found that a progesterone concentration of 10^−9^ M significantly increased the activities of SOD, POD, and CAT during bean (*Phaseolus vulgaris*) seed germination [47]. Similar results were obtained in maize (*Zea mays*), where progesterone (10^−4^–10^−15^ M) significantly increased the germination rate of maize seeds and enhanced root and coleoptyle length, while the activities of α-amylase, SOD, CAT, peroxidase (POX), and polyphenol oxidase (PPO) were simultaneously induced [48]. Further studies in maize showed that progesterone accelerated seed germination by activating mitochondrial respiration and related pathways, and genes (such as *CS*, *COX19*, *Pdh1*, and *ATP6*) related to mitochondrial respiration were upregulated [49]. Thus, the promoting effect of progesterone on seed germination might potentially be related to antioxidant enzyme activation, α-amylase, and mitochondrial respiration.

### 4.3. Reproductive Development

Endogenous progesterone content in kiwifruit (*Actinidia deliciosa*) pollen increases gradually with pollen germination [50]. Exogenous progesterone application significantly stimulated pollen germination and tube elongation, and thereby strongly enhanced tobacco (*Nicotiana tabacum*) male gametophyte growth [51]. Progesterone clearly not only regulates plant vegetative growth, but also has certain effects on reproductive development. Studies in both wheat and *Arabidopsis* confirmed that progesterone induced plant flowering and promoted reproductive growth [53,54,55]. Additionally, the gene *CYP11A1* in the bovine adrenal cortex, which catalyzes the conversion of cholesterol into pregnenolone, was transformed into tobacco, and *CYP11A1* overexpression-transgenic plants had shortened vegetative periods (early flowering and maturation of bolls) [52,56]. Therefore, the present findings imply that appropriate concentrations of progesterone may drive reproductive processes by promoting pollen germination and growth.

## 5. Plant Biotic and Abiotic Stress Regulation by Progesterone

Although progesterone is not yet recognized as a plant hormone, it has similar functions to plant hormones, which mediate plant growth and development and also regulate biotic and abiotic stress (Table 2, Figure 3).

### 5.1. Salt Stress

Progesterone at an appropriate concentration can increase the activities of SOD, POD, CAT, ascorbate peroxidase (APX), and nitrate reductase (NR), and reduce lipid peroxidation and hydrogen peroxide (H_2_O_2_) content, thereby alleviating salt-induced inhibition of wheat seedling growth [57]. Improved plant salt tolerance by progesterone was also confirmed in *Phaseolus vulgaris*, *Zea mays*, and *Poa pratensis*, and, in addition to increasing antioxidant enzyme activity to alleviate salt stress, progesterone can ease the salt-reduced K/Na ratio and pigment content (including chlorophyll and carotenoids) [58,59,60].

### 5.2. Chilling Stress

In chickpea, certain oxidative stress indicators (such as superoxide production, electrolyte leakage, H_2_O_2_, and MDA content) induced by chilling stress can be lessened by progesterone through increased relative leaf water and chlorophyll content, as well as antioxidant enzyme activities, and the application of progesterone lowered the freezing point of chickpea seedlings from −4 to −5.5 °C. These physiological changes contribute to the protective effect of progesterone on chickpeas when exposed to chilling stress [61]. Studies in maize suggested that the protective effect of progesterone against cold injury was also related to its modulation of the mitochondrial respiratory pathway (MRP). Cold stress activated the cytochrome pathway (CP), alternative respiratory pathway (AP), and total cellular respiratory rate (TCR), which can be further enhanced under progesterone pretreatment. Moreover, both the transcript and protein levels of AOX (the terminal oxidase in the AP) increased due to progesterone application [62]. Under the chilling stress, progesterone can reduce the generation of O_2_^•^^−^ and H_2_O_2_, and upregulate the transcription and protein levels of AOX in postharvest bananas (*Musa nana Lour*). Notably, the antioxidant system, activated by progesterone, also played a crucial role in chilling injury protection [63]. Similar results were also found in postharvest sweet potato (*Ipomoea batatas*) tuberous roots [64]. Therefore, the alternative respiratory pathway, especially the *AOX* gene, is involved in the alleviating effect of progesterone on chilling stress. Additionally, the protective effect of progesterone on wheat seedlings exposed to low temperature was related to its effects on lipid membrane structures [65].

### 5.3. Drought Stress

Janeczko et al. [41] found that drought stress decreased progesterone content in drought-sensitive wheat cultivars (Cv. Katoda), but increased progesterone content in drought-tolerant wheat cultivars (cv. Monsun). Moreover, cell membrane progesterone binding sites increased due to drought stress in Katoda, but not in Monsun, while progesterone binding sites in the cytoplasm increased due to drought in Monsun, but not in Katoda. These results indicate that progesterone responds to drought stress. A study of tomato plants that overexpressed mammalian *CYP11A1* revealed that the progesterone content in transgenic plants was 3–5 times higher compared to wild-type plants, and that transgenic plants were more resistant to drought and long-term dehydration [56].

### 5.4. Heat and High Light

Heat and high light often become abiotic stresses that limit wheat growth. Progesterone can effectively protect wheat from oxidative damage caused by heat and high light by activating the antioxidant system and repairing photosystem II (PSII) [66,67].

### 5.5. Biotic Stresses

Progesterone diminished necrotic symptoms and electrolyte leakage caused by *Pseudomonas* bacteria in *Arabidopsis* leaves [68]. Mammalian *CYP11A1*-overexpressing transgenic tobacco plants exhibited resistance to infection by the fungal pathogen *Botrytis cinerea*, and the average leaf affected area of transgenic plants was about 12 times smaller than non-transgenic plants [56].

In general, the current research on the regulation of progesterone on plant biotic/abiotic stress responses mainly focuses on its effects on traditional stress indicators, such as ROS content, antioxidant enzyme activities, photosynthesis, and respiration rate. However, little is known about how progesterone transmits signals into cells to regulate physiological processes, and the key regulatory genes in the progesterone signaling pathway are barely identified. In addition, plant hormones are recognized to be involved in plant stress regulation, and whether there is a correlation between progesterone and these stress hormones has not yet been studied. There is still a long way ahead to explore the molecular regulation mechanism of progesterone in response to plant biotic/abiotic stress.

## 6. The Regulation of Progesterone on Photosynthesis

Photosynthesis is the basis of crop yield, and progesterone is known to play an important role in the regulation of photosynthesis, which is mainly reflected by three aspects: (1) Improved chlorophyll content—progesterone can significantly increase the chlorophyll content of wheat leaves under salt stress [57], and improve low-temperature effects on the chlorophyll content of chickpea seedlings [61]. (2) Protecting photosystem activity—chlorophyll fluorescence parameters decreased significantly in *Arabidopsis* leaves infected with *Pseudomonas syringae*, but this inhibition was partially restored by progesterone [68]. The damage caused by heat and high light on PSII activity can be alleviated by progesterone in wheat leaves, and this protective effect may be related to accelerating D1 protein phosphorylation [66,67]. Wheat studies showed that progesterone can increase the electron energy flux in the PSII by 12% under drought stress [54]. (3) Increased Rubisco (CO_2_ binding enzyme) activity—trilostane, a progesterone synthesis inhibitor, significantly decreased Rubisco activity in wheat leaves under drought stress, but its activity was restored by the application of exogenous progesterone [54]. The increase of photosynthesis is conducive to the formation of crop yield, so the protection and promotion of progesterone on photosynthesis will be the gospel of crop yield improvement. The application of progesterone as a growth regulator in crop cultivation can be considered in follow-up studies.

## 7. Correlation between Progesterone and Brassinosteroid

Both progesterone and brassinosteroid (BR) are steroids. Firstly, progesterone and BR are cross-linked in metabolic processes. Deletion of the steroid 5α-reductase DET2, which can reduce progesterone to 5α-pregnane-3,20-dione, resulted in decreased BR accumulation in *Arabidopsis* [69]. Secondly, progesterone and BR are related in the regulation of hypocotyl elongation. Exogenous BR can promote or inhibit hypocotyl growth, depending on its concentration and light conditions [70], but overexpression or deletion of *MSBP1* (a progesterone receptor) alters *Arabidopsis* plant sensitivity to BR [36]. Overexpression of *MSBP1* inhibits cell expansion and BR responses, but this phenomenon is restored by co-overexpression of *BAK1* in *Arabidopsis*; MSBP1 could specifically interact with the extracellular domain of BAK1 in vivo, and thus negatively regulates BR signaling [37] (Figure 2). In the regulation of wheat reproductive development, progesterone and BR functions are opposed to one another; that is, progesterone promotes the transition from vegetative growth to reproduction and heading, while BR inhibits this [54].

## 8. Conclusions and Prospects

Plant progesterone research lags far behind its contemporary animal studies. This review traced the discovery of progesterone in plants and the study of its metabolism. Progesterone is ubiquitous in plants at low concentrations, and we now have a relatively clear understanding of its membrane receptor (MSBP1). Secondly, this paper summarized the regulatory role of progesterone in plant growth, development, and stress responses. These properties of progesterone are very similar to plant hormones [71], and therefore it is expected to be a phytohormone candidate.

Although plant progesterone has gradually attracted scholarly attention, and its metabolism and physiological functions have been increasingly explored, many unsolved mysteries remain. For example, the side-chain-cleavage processes of the progesterone precursor and its related enzymes have not yet been identified. Progesterone has a binding site in the cytoplasm, so what is the progesterone receptor in the cytoplasm? What is the mechanism between progesterone and its membrane receptor (MSBP1) to regulate downstream physiological functions? Apart from the above-mentioned, does progesterone have other physiological functions, and are these regulatory functions generally applicable to the plant kingdom? These questions require more research. Moreover, from previous studies on the regulation of plant growth, development, and stress response by progesterone, our understanding of the regulatory functions of progesterone generally remains at the physiological level, and rarely involves molecular mechanisms. More studies on progesterone receptors and response genes will thus greatly contribute to our understanding of the progesterone signaling pathway. Based on this, we reviewed plant progesterone in the hope of attracting the attention of more researchers, and to promote the current understanding of progesterone in plants.

## Figures and Tables

**Table 1:** The regulation of progesterone on plant growth and development.

Plant Growth and Development	Progesterone Action	Plant Species	Reference
Shoot and root growth	Progesterone regulated plant growth in a concentration dependent manner.	*Arabidopsis thaliana*	[11]
		*Helianthus annuus*	[42]
		*Cicer arietinum*	[43]
Tissue culture	Progesterone improved shoot and callus formation.	*Onobrychis sativa*	[44]
	Progesterone regulated responded embryogenic callus and regenerable callus induction.	*Triticum aestivum*	[45]
Seed germination	Progesterone accelerated seed germination.	*Cicer arietinum*	[46]
		*Phaseolus vulgaris*	[47]
		*Zea mays*	[48,49]
Reproductive development	Progesterone increased gradually with pollen germination.	*Actinidia deliciosa*	[50]
	Progesterone stimulated pollen germination and tube elongation.	*Nicotiana tabacum*	[51,52]
	Progesterone induced plant flowering and promoted reproductive growth.	*Triticum aestivum*	[53,54]
		*Arabidopsis thaliana*	[55]

**Table 2:** The alleviating effects of progesterone on biotic and abiotic stresses in plants.

Biotic/Abiotic Stress	Progesterone Action	Plant Species	Reference
Salt	Progesterone stimulated enzymatic and non-enzymatic antioxidant mechanisms and increased the levels of osmoprotectants.	*Triticum aestivum*	[57]
	Progesterone stimulated SOD, POX, and CAT activities and mitigated the salt-reduced K/Na ratio.	*Phaseolus vulgaris*	[58]
	Progesterone stimulated antioxidant activity and osmoprotectants accumulation.	*Zea mays*	[59]
	Progesterone improved salinity tolerance and increased pigments and antioxidant enzyme activities.	*Poa pratensis*	[60]
Chilling	Progesterone improved relative leaf water content, chlorophyll content, and antioxidative activity.	*Cicer arietinum*	[61]
	Progesterone enhanced mitochondrial respiratory pathway, and upregulated the transcript level and protein accumulation of alternative oxidase (AOX).	*Zea mays*	[62]
	Progesterone induced AOX and improved enzyme and non-enzymatic antioxidant defenses.	*Musa nana Lour*	[63]
	Progesterone enhanced the transcription level of IbAOX1 and the activity of AOX, inhibited the formation of chilling injury, reduced membrane permeability, malonaldehyde levels, and ROS production, and enhanced the antioxidant protection system.	*Ipomoea batatas*	[64]
	Progesterone increased the area per lipid molecule in monolayers, resulting in formation of more flexible surface structures.	*Triticum aestivum*	[65]
Drought	Progesterone-binding sites on the cell membrane were increased by drought stress in drought-sensitive cultivar (Katoda) but not in drought-tolerant cultivar (Monsun), while progesterone-binding sites in the cytoplasm were increased by drought in Monsun but not in Katoda.	*Triticum aestivum*	[41]
	Overexpressing mammalian CYP11A1 in tomato can significantly increase tolerance to drought and long-term dehydration.	*Solanum lycopersicum*	[56]
Heat and high light	Progesterone alleviated heat-stress-induced hydrogen peroxide, malondialdehyde, and relative electrolytic leakage, improved the activities of superoxide dismutase, catalase, and peroxidase, and reduced PSII injury by promoting D1 protein phosphorylation.	*Triticum aestivum*	[66]
	Progesterone enhanced antioxidant defense system and facilitated D1 protein stability under heat and high light cross-stress.	*Triticum aestivum*	[67]
Biotic stresses	Progesterone diminished the necrotic symptoms and the electrolyte leakage, and improved the efficiency of photosystem II caused by *Pseudomonas bacteria.*	*Arabidopsis thaliana*	[68]
	CYP11A1-overexpressing transgenic tobacco exhibited resistance to infection by fungal pathogens *Botrytis cinerea.*	*Nicotiana tabacum*	[56]

## Data Availability

Not applicable.

## References

[1] Gompel A. (2020). Progesterone and endometrial cancer. Best Pract. Res. Clin. Obstet. Gynaecol..

[2] Trabert B., Sherman M.E., Kannan N., Stanczyk F.Z. (2020). Progesterone and breast cancer. Endocr. Rev..

[3] González-Orozco J.C., Camacho-Arroyo I. (2019). Progesterone actions during central nervous system development. Front. Neurosci..

[4] Guennoun R. (2020). Progesterone in the brain: Hormone, neurosteroid and neuroprotectant. Int. J. Mol. Sci..

[5] Brotfain E., Gruenbaum S.E., Boyko M., Kutz R., Zlotnik A., Klein M. (2016). Neuroprotection by estrogen and progesterone in traumatic brain injury and spinal cord injury. Curr. Neuropharmacol..

[6] Wright D.W., Yeatts S.D., Silbergleit R. (2015). Progesterone in traumatic brain injury. N. Engl. J. Med..

[7] Gawienowski A.M., Gibbs C.C. (1968). Identification of cholesterol and progesterone in apple seeds. Steroids.

[8] Šaden-Krehula M., Tajić M., Kolbah D. (1979). Sex hormones and corticosteroids in pollen of *Pinus nigra*. Phytochemistry.

[9] Carson J.D., Jenkins R.L., Wilson E.M., Howell W.M., Moore R. (2008). Naturally occurring progesterone in loblolly pine (*Pinus taeda* L.): A major steroid precursor of environmental androgens. Environ. Toxicol. Chem. Int. J..

[10] Simons R., Grinwich D. (1989). Immunoreactive detection of four mammalian steroids in plants. Can. J. Bot..

[11] Iino M., Nomura T., Tamaki Y., Yamada Y., Yoneyama K., Takeuchi Y., Mori M., Asami T., Nakano T., Yokota T. (2007). Progesterone: Its occurrence in plants and involvement in plant growth. Phytochemistry.

[12] Simerský R., Novák O., Morris D.A., Pouzar V., Strnad M. (2009). Identification and quantification of several mammalian steroid hormones in plants by UPLC-MS/MS. J. Plant Growth Regul..

[13] Pauli G.F., Friesen J.B., Gödecke T., Farnsworth N.R., Glodny B. (2010). Occurrence of progesterone and related animal steroids in two higher plants. J. Nat. Prod..

[14] Bennett R., Heftmann E., Winter B.J. (1969). Conversion of sitosterol to progesterone by *Digitalis lanata*. Die Nat..

[15] Stohs S., El-Olemy M. (1971). Pregnenolone and progesterone from 20α-hydroxycholesterol by *Cheiranthus cheiri* leaf homogenates. Phytochemistry.

[16] Lindemann P., Luckner M. (1997). Biosynthesis of pregnane derivatives in somatic embryos of *Digitalis lanata*. Phytochemistry.

[17] Sundararaman P., Djerassi C. (1977). A convenient synthesis of progesterone from stigmasterol. J. Org. Chem..

[18] Janeczko A. (2012). The presence and activity of progesterone in the plant kingdom. Steroids.

[19] Aringer L., Eneroth P., Nordström L. (1979). Side-chain cleavage of 4-cholesten-3-one, 5-cholesten-3α-ol, β-sitosterol, and related steroids in endocrine tissues from rat and man. J. Steroid Biochem..

[20] Hoyte R., RB H. (1979). Enzymatic side chain cleavage of c-20 alkyl and aryl analogs of (20-s)-20-hydroxycholesterol: Implications for the biosynthesis of pregnenolone. J. Biol. Chem..

[21] Lindemann P. (2015). Steroidogenesis in plants–Biosynthesis and conversions of progesterone and other pregnane derivatives. Steroids.

[22] Seidel S., Kreis W., Reinhard E. (1990). D5-3b-Hydroxysteroid dehydrogenase/D5-D4-ketosteroid isomerase (3b-HSD), a possible enzyme of cardiac glycoside biosynthesis, in cell cultures and plants of *Digitalis lanata* EHRH. Plant Cell Rep..

[23] Gärtner D.E., Keilholz W., Seitz H.U. (1994). Purification, characterization and partial peptide microsequencing of progesterone 5β-reductase from shoot cultures of *Digitalis purpurea*. Eur. J. Biochem..

[24] Herl V., Fischer G., Müller-Uri F., Kreis W. (2006). Molecular cloning and heterologous expression of progesterone 5β-reductase from *Digitalis lanata* Ehrh. Phytochemistry.

[25] Gärtner D.E., Wendroth S., Seitz H.U. (1990). A stereospecific enzyme of the putative biosynthetic pathway of cardenolides: Characterization of a progesterone 5β-reductase from leaves of *Digitalis purpurea* L.. FEBS Lett..

[26] Caspi E., Lewis D. (1967). Progesterone: Its possible role in the biosynthesis of cardenolides in *Digitalis lanata*. Science.

[27] Klein J., Horn E., Ernst M., Leykauf T., Leupold T., Dorfner M., Wolf L., Ignatova A., Kreis W., Munkert J. (2021). RNAi-mediated gene knockdown of progesterone 5β-reductases in *Digitalis lanata* reduces 5β-cardenolide content. Plant Cell Rep..

[28] Herl V., Fischer G., Reva V., Stiebritz M., Muller Y., Müller-Uri F., Kreis W. (2009). The VEP1 gene (At4g24220) encodes a short-chain dehydrogenase/reductase with 3-oxo-Δ4, 5-steroid 5β-reductase activity in *Arabidopsis thaliana* L.. Biochimie.

[29] Kairuz E., Pérez-Alonso N., Capote-Pérez A., Pérez-Pérez A., Espinosa-Antón A.A., Angenon G., Jiménez E., Chong-Pérez B. (2020). Enhancement of cardenolide production in transgenic *Digitalis purpurea* L. by expressing a progesterone-5β-reductase from *Arabidopsis thaliana* L.. Ind. Crops Prod..

[30] Bauer P., Munkert J., Brydziun M., Burda E., Muller-Uri F., Groger H., Muller Y., Kreis W. (2010). Highly conserved progesterone 5b-reductase genes (P5bR) from 5b-cardenolide-free and 5b-cardenolide-producing angiosperms. Phytochemistry.

[31] Pérez-Bermúdez P., Moya Garcia A.A., Tuñón I., Gavidia I. (2010). *Digitalis purpurea* P5βR2, encoding steroid 5β-reductase, is a novel defense-related gene involved in cardenolide biosynthesis. New Phytol..

[32] Wendroth S., Seitz H.U. (1990). Characterization and localization of progesterone 5 α-reductase from cell cultures of foxglove (Digitalis *lanata* EHRH). Biochem. J..

[33] Li J., Biswas M.G., Chao A., Russell D.W., Chory J. (1997). Conservation of function between mammalian and plant steroid 5α-reductases. Proc. Natl. Acad. Sci. USA.

[34] Rosati F., Bardazzi I., De Blasi P., Simi L., Scarpi D., Guarna A., Serio M., Racchi M.L., Danza G. (2005). 5α-Reductase activity in Lycopersicon esculentum: Cloning and functional characterization of LeDET2 and evidence of the presence of two isoenzymes. J. Steroid Biochem. Mol. Biol..

[35] Rosati F., Danza G., Guarna A., Cini N., Racchi M.L., Serio M. (2003). New evidence of similarity between human and plant steroid metabolism: 5α-reductase activity in *Solanum malacoxylon*. Endocrinology.

[36] Yang X.-H., Xu Z.-H., Xue H.-W. (2005). Arabidopsis membrane steroid binding protein 1 is involved in inhibition of cell elongation. Plant Cell.

[37] Song L., Shi Q.-M., Yang X.-H., Xu Z.-H., Xue H.-W. (2009). Membrane steroid-binding protein 1 (MSBP1) negatively regulates brassinosteroid signaling by enhancing the endocytosis of BAK1. Cell Res..

[38] Shi Q.-M., Yang X., Song L., Xue H.-W. (2011). Arabidopsis MSBP1 is activated by HY5 and HYH and is involved in photomorphogenesis and brassinosteroid sensitivity regulation. Mol. Plant.

[39] Yang X., Song L., Xue H.-W. (2008). Membrane steroid binding protein 1 (MSBP1) stimulates tropism by regulating vesicle trafficking and auxin redistribution. Mol. Plant.

[40] Janeczko A., Budziszewska B., Skoczowski A., Dybała M. (2008). Specific binding sites for progesterone and 17beta-estradiol in cells of *Triticum aestivum* L.. Acta Biochim. Pol..

[41] Janeczko A., Oklešťková J., Siwek A., Dziurka M., Pociecha E., Kocurek M., Novák O. (2013). Endogenous progesterone and its cellular binding sites in wheat exposed to drought stress. J. Steroid Biochem. Mol. Biol..

[42] Bhattacharya B., Gupta K. (1981). Steroid hormone effects on growth and apical dominance of sunflower. Phytochemistry.

[43] Erdal S., Dumlupinar R. (2011). Mammalian sex hormones stimulate antioxidant system and enhance growth of chickpea plants. Acta Physiol. Plant..

[44] Dadaşoğlu E., Tosun M. (2018). The effect of mammalian sex hormones on in vitro properties of sainfoin (*Onobrychis sativa* L.). Fresenius Environ. Bull..

[45] Turkoglu A. (2022). Effects of mammalian sex hormones on regeneration capacity, retrotransposon polymorphism and genomic instability in wheat (*Triticum aestivum* L.).

[46] Erdal S., Dumlupinar R. (2010). Progesterone and β-estradiol stimulate seed germination in chickpea by causing important changes in biochemical parameters. Z. Nat. C.

[47] Erdal S. (2009). Effects of mammalian sex hormones on antioxidant enzyme activities, H2O2 content and lipid peroxidation in germinating bean seeds. J. Fac. Agric..

[48] Erdal S., Dumlupinar R., Cakmak T., Genisel M. (2010). Mammalian sex hormones influence germination velocity and enzyme activities in germinating maize seeds. Fresenius Environ. Bull..

[49] Turk H. (2021). Progesterone Promotes Mitochondrial Respiration at the Biochemical and Molecular Level in Germinating Maize Seeds. Plants.

[50] Speranza A., Crosti P., Malerba M., Stocchi O., Scoccianti V. (2011). The environmental endocrine disruptor, bisphenol A, affects germination, elicits stress response and alters steroid hormone production in kiwifruit pollen. Plant Biol..

[51] Ylstra B., Touraev A., Brinkmann A.O., Heberle-Bors E., Tunen A. (1995). Steroid hormones stimulate germination and tube growth of in vitro matured tobacco pollen. Plant Physiol..

[52] Spivak S., Berdichevets I., Yarmolinsky D., Maneshina T., Shpakovski G., Kartel N. (2009). Construction and characteristics of transgenic tobacco *Nicotiana tabacum* L. plants expressing CYP11A1 cDNA encoding cytochrome P450SCC. Russ. J. Genet..

[53] Janeczko A., Filek W. (2002). Stimulation of generative development in partly vernalized winter wheat by animal sex hormones. Acta Physiol. Plant..

[54] Janeczko A., Oklestkova J., Novak O., Śniegowska-Świerk K., Snaczke Z., Pociecha E. (2015). Disturbances in production of progesterone and their implications in plant studies. Steroids.

[55] Janeczko A., Filek W., Biesaga-Kościelniak J., Marcińska I., Janeczko Z. (2003). The influence of animal sex hormones on the induction of flowering in *Arabidopsis thaliana*: Comparison with the effect of 24-epibrassinolide. Plant Cell Tissue Organ Cult..

[56] Shpakovski G.V., Spivak S.G., Berdichevets I.N., Babak O.G., Kubrak S.V., Kilchevsky A.V., Aralov A.V., Slovokhotov I.Y., Shpakovski D.G., Baranova E.N. (2017). A key enzyme of animal steroidogenesis can function in plants enhancing their immunity and accelerating the processes of growth and development. BMC Plant Biol..

[57] Erdal S. (2012). Alleviation of salt stress in wheat seedlings by mammalian sex hormones. J. Sci. Food Agric..

[58] Erdal S., Genisel M., Turk H., Gorcek Z. (2012). Effects of progesterone application on antioxidant enzyme activities and K+/Na+ ratio in bean seeds exposed to salt stress. Toxicol. Ind. Health.

[59] Erdal S. (2012). Exogenous mammalian sex hormones mitigate inhibition in growth by enhancing antioxidant activity and synthesis reactions in germinating maize seeds under salt stress. J. Sci. Food Agric..

[60] Sabzmeydani E., Sedaghathoor S., Hashemabadi D. (2021). Effect of salicylic acid and progesterone on physiological characteristics of *Kentucky bluegrass* under salinity stress. Rev. Cienc. Agrícolas.

[61] Genisel M., Turk H., Erdal S. (2013). Exogenous progesterone application protects chickpea seedlings against chilling-induced oxidative stress. Acta Physiol. Plant..

[62] Erdal S., Genisel M. (2016). The property of progesterone to mitigate cold stress in maize is linked to a modulation of the mitochondrial respiratory pathway. Theor. Exp. Plant Physiol..

[63] Hao J., Li X., Xu G., Huo Y., Yang H. (2019). Exogenous progesterone treatment alleviates chilling injury in postharvest banana fruit associated with induction of alternative oxidase and antioxidant defense. Food Chem..

[64] Chen H., Zhou S., Li X., Yang H. (2022). Exogenous progesterone alleviates chilling injury by upregulating IbAOX1 to mediate redox homeostasis and proline accumulation in postharvest sweetpotato tuberous root. Postharvest Biol. Technol..

[65] Filek M., Rudolphi-Skórska E., Sieprawska A., Kvasnica M., Janeczko A. (2017). Regulation of the membrane structure by brassinosteroids and progesterone in winter wheat seedlings exposed to low temperature. Steroids.

[66] Xue R., Wang S., Xu H., Zhang P., Li H., Zhao H. (2017). Progesterone increases photochemical efficiency of photosystem II in wheat under heat stress by facilitating D1 protein phosphorylation. Photosynthetica.

[67] Su X., Wu S., Yang L., Xue R., Li H., Wang Y., Zhao H. (2014). Exogenous progesterone alleviates heat and high light stress-induced inactivation of photosystem II in wheat by enhancing antioxidant defense and D1 protein stability. Plant Growth Regul..

[68] Janeczko A., Tóbiás I., Skoczowski A., Dubert F., Gullner G., Barna B. (2013). Progesterone moderates damage in *Arabidopsis thaliana* caused by infection with Pseudomonas syringae or P. fluorescens. Biol. Plant..

[69] Fujioka S., Li J., Choi Y.-H., Seto H., Takatsuto S., Noguchi T., Watanabe T., Kuriyama H., Yokota T., Chory J. (1997). The Arabidopsis deetiolated2 mutant is blocked early in brassinosteroid biosynthesis. Plant Cell.

[70] Li J., Nagpal P., Vitart V., McMorris T.C., Chory J. (1996). A role for brassinosteroids in light-dependent development of Arabidopsis. Science.

[71] Davies P.J. (2010). The plant hormones: Their nature, occurrence, and functions. Plant Hormones.

